# Crystal structure of hepta­kis­(2,6-di­methyl­phenyl isocyanide-κ*C*)vanadium(I) iodide

**DOI:** 10.1107/S2056989015006015

**Published:** 2015-03-28

**Authors:** Mikhail E. Minyaev, John E. Ellis

**Affiliations:** a A.V. Topchiev Institute of Petrochemical Synthesis, Russian Academy of Sciences, 29 Leninsky Prospect, 119991, Moscow, Russian Federation; b University of Minnesota, 207 Pleasant Str. SE, Minneapolis, MN 55455, USA

**Keywords:** crystal structure, homoleptic seven-coordinate vanadium(I) complex, isocyanide, iodide,

## Abstract

The title salt, [V(C_9_H_9_N)_7_]^+^I^−^ or [V(CNX­yl)_7_]^+^I^−^ (Xyl is 2,6-di­methyl­phen­yl), crystallized from tetra­hydro­furan at low temperatures after reacting (Et_4_N)^+^[V(CO)_6_]^−^, excess of CNXyl and iodine. The complex cation and the two crystallographically different iodide anions, each located on a different glide plane, are well separated in the crystal structure. The V(CN)_7_ core of the cation has the form of a distorted monocapped trigonal prism. This compound is of inter­est as the first isolable homoleptic seven-coordinate vanadium analog of the 18-electron [V(CO)_7_]^+^ monocation.

## Abstract 2

The complex cation and the two crystallographically different iodide anions, each located on a different glide plane, are well separated in the crystal structure. The V(CN)_7_ core of the cation has the form of a distorted monocapped trigonal prism. This compound is of inter­est as the first isolable homoleptic seven-coordinate vanadium analog of the 18-electron [V(CO)_7_]^+^ monocation.

## Chemical context   

**Figure 1:**
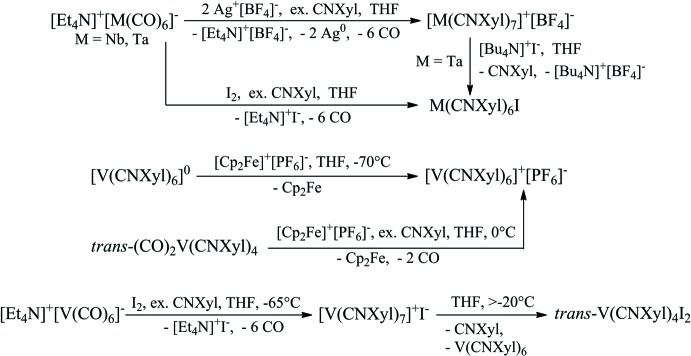
Scheme showing preparation and transformations of some isocyanide complexes of group 5 metals.

Reaction of the carbonyl­ate anion [*M*(CO)_6_]^−^ (*M* = Nb, Ta) with an Ag^+^ cation and excess of xylyl isocyanide (CNX­yl) leads to formation of the 18-electron cation [*M*(CNX­yl)_7_]^+^ (Fig. 1 ▸, see: Barybin *et al.*, 2007 ▸). However, oxidation of V(CNX­yl)_6_ (Barybin *et al.*, 1998 ▸, 2000 ▸) or of *trans-*(CO)_2_V(CNX­yl)_4_ in the presence of excess CNXyl (Barybin *et al.*, 2000 ▸) with the ferrocenyl cation provides the stable 16-electron cation [V(CNX­yl)_6_]^+^ (Fig. 1 ▸). Also of inter­est are observations of [*M*(CO)_7_]^+^ (*M* = Nb, Ta) species in the gas phase and unsuccessful attempts to detect [V(CO)_7_]^+^ under the same conditions (Ricks *et al.*, 2009 ▸). On this basis, isolation of the title compound, [V(CNX­yl)_7_]^+^I^−^, was a totally unexpected result. Only one homoleptic seven-coordinate vanadium complex with only monodentate ligands has been previously reported, *viz.* K_4_[V(CN)_7_]·2H_2_O (Levenson & Towns, 1974 ▸).

Oxidation of [*M*(CO)_6_]^−^ (*M* = Nb, Ta) with one equivalent of I_2_ in the presence of excess CNXyl gives the 18-electron uncharged mol­ecular complexes *M*(CNX­yl)_6_I bearing only six isocyanide ligands (Fig.1, see: Barybin *et al.*, 2007 ▸). Anion-exchange reaction between [Ta(CNX­yl)_7_]^+^[BF_4_]^−^ and [Bu_4_N]^+^I^−^ leads to the loss of one CNXyl ligand and to formation of Ta(CNX­yl)_6_I (Barybin *et al.*, 2007 ▸). Rehder *et al.* (1999 ▸) have isolated only *trans-*V(CNX­yl)_4_I_2_ being formed in a similar oxidation reaction from [V(CO)_6_]^−^. We report herein that the 18-electron inter­mediate ionic complex [V(CNX­yl)_7_]^+^I^−^, which is formed and stable at low temperatures, can be isolated from the last reaction (Fig. 1 ▸). It is soluble in THF but insoluble in toluene. At room temperature in THF, it completely decomposes during seven to ten days to produce *trans-*V(CNX­yl)_4_I_2_ (based on X-ray, NMR and IR data), free CNXyl and V(CNX­yl)_6_ (based on NMR and IR studies).
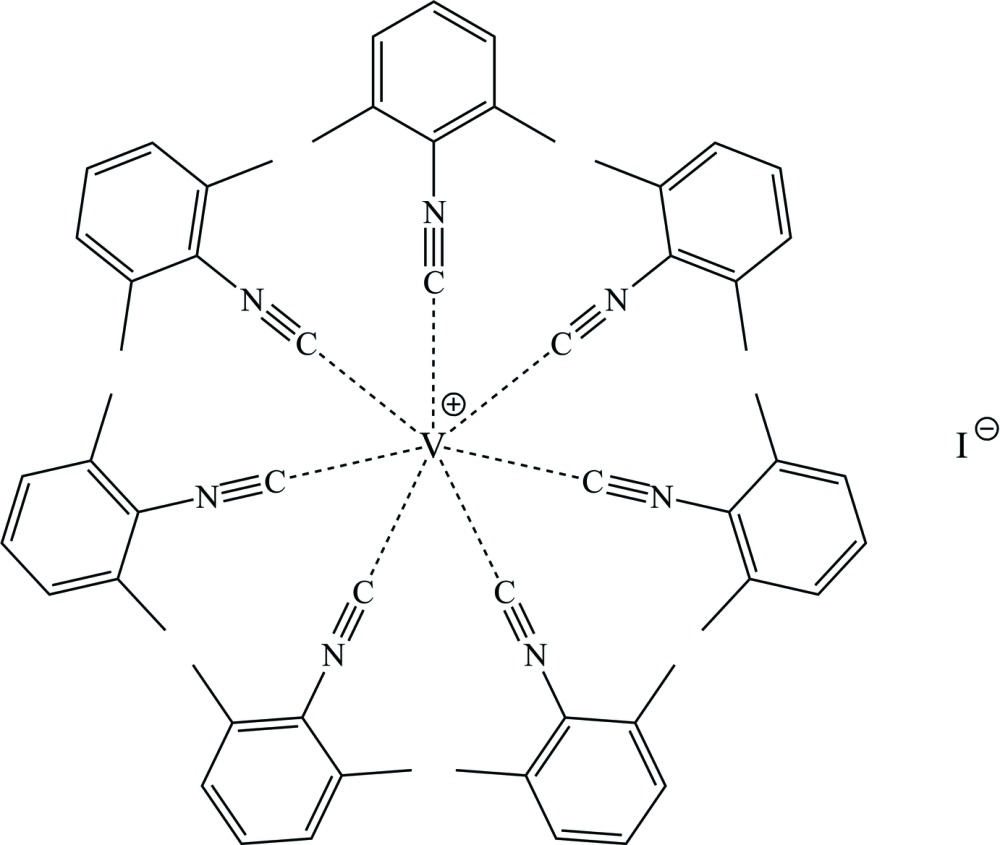


## Structural commentary   

**Figure 2:**
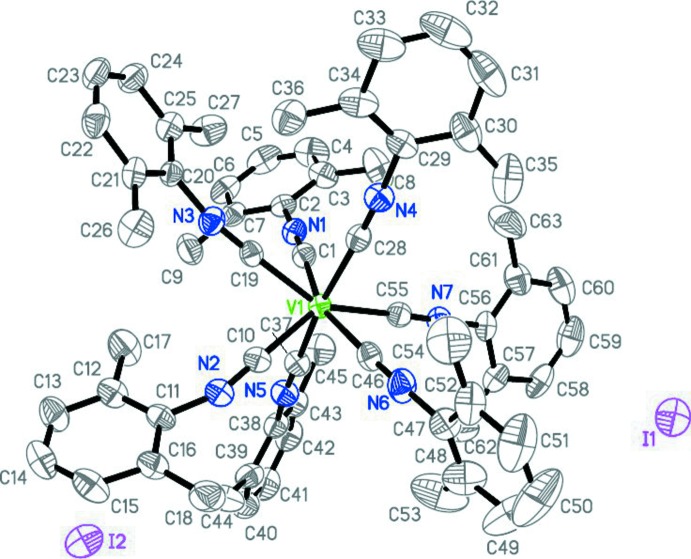
The mol­ecular structure of [V(CNX­yl)_7_]^+^I^−^ with displacement parameters drawn at the 50% probability level. Hydrogen atoms are omitted for clarity.

The cation and anion in the title compound are separated in the crystal structure. The asymmetric unit contains an unusual seven-coordinate vanadium(I) cation, [V(CNX­yl)_7_]^+^, and two iodide anions, each of which is located on a different glide plane (Fig. 2 ▸). No solvent mol­ecule is present regardless of potentially solvent-accessible volumes of 49 Å^3^. There are some non-valent short contacts: I⋯H—C_Ar_ (I1⋯H51*A* 3.091 Å), C_Ar_—H⋯C_Ar_ (H13*A*⋯C43 2.760, H60*A*⋯C40 2.898 Å), CH_2_—H⋯C_Ar_ (H8*C*⋯C14 2.831, H27*A*⋯C21 2.770, H36*A*⋯C57 2.887, H36*A*⋯C58 2.765, H45*A*⋯C34 2.812 Å), CH_2_—H⋯CH_3_ (H27*A*⋯C26 2.878 Å), C_Ar_—H⋯CH_3_ (H41*A*⋯C18 2.857 Å). However, no significant inter­ionic inter­actions are present.

**Figure 3:**
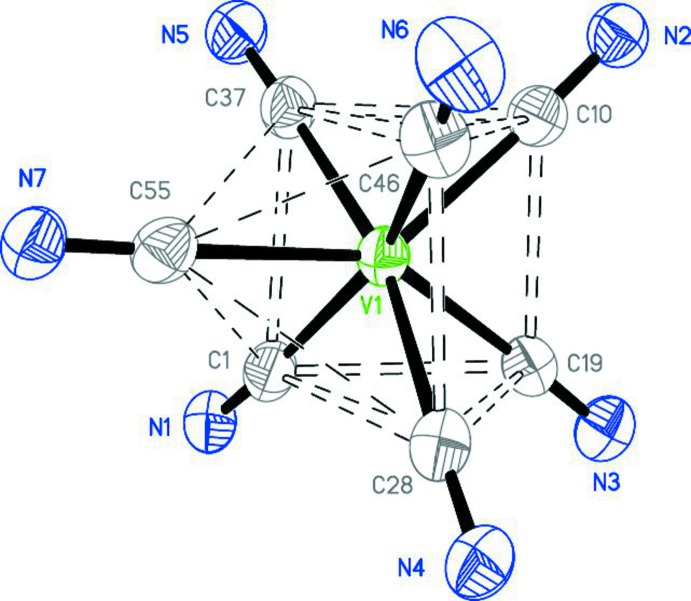
The V(CN)_7_ core of the cation is a monocapped trigonal prism. 2,6-Di­methyl­phenyl groups are not shown.

The coordination polyhedron of the [V(CNX­yl)_7_]^+^ cation is a distorted monocapped trigonal prism (Fig. 3 ▸), supported by calculations with the *HEPTA* program (Maseras & Eisenstein, 1997 ▸). Deviations (dimensionless) from three ideal geometries have been calculated using 21 real and optimal inter­ligand angles (Maseras & Eisenstein, 1997 ▸). The lowest deviations are 3.57 for a capped trigonal prism (*C*_2*v*_) with C55 N7-Xyl as a capping ligand, 5.69 for a capped octa­hedron (*C*_3*v*_) with C10 N2-Xyl as a capping ligand, and 13.86 for a penta­gonal bipyramid (*D*_5*h*_) with the C28 N4-Xyl and C37 N5-Xyl ligands being in the axial positions.

The V—C distances vary from 2.002 (4) to 2.062 (4) Å, with the exception for the capping ligand which is associated with the longest bond, V1—C55 = 2.107 (3) Å. For the six ligands, the C N triple-bond lengths lie in a very narrow inter­val from 1.160 (4) to 1.165 (4) Å. The value for the capping ligand is 1.152 (4) Å (C55—N7). At the same time, all C N distances are about the same, as in most free isocyanides (1.14 to 1.16 Å) found in the Cambridge Structural Database (Groom & Allen, 2014 ▸). The V—C N angles are nearly linear (see: Fig. 3 ▸), having values between 175.7 (3) and 177.7 (3)°. The isocyanide ligands are slightly bent about the nitro­gen atoms with the C N—C angles between 161.0 (3) and 178.1 (4)°. It should be noted that the C N bond length and the C N—C angle of the capping ligand (C55 N7—X­yl) correspond to a nearly unperturbed isocyanide mol­ecule.

## Database survey   

According to the Cambridge Structural Database (CSD version 5.35 with updates, Groom & Allen, 2014 ▸), the number of group 5 metal isocyanide compounds structurally determined is limited to 65. Among them, there are only ten crystal structures of cationic and/or halogen-containing isocyanide complexes related to the present work like [*M*(CN*R*)_*x*_]^*n*+^ or *M*(CN*R*)_*x*_Hal_*y*_ (*M* = V, Nb, Ta), which do not include any other ligands: Three isocyanide complexes represented by a 16-electron vanadium(I) cation in [V(CNX­yl)_6_]^+^[PF_6_]^−^(THF) (CSD refcodes PERCAQ; Barybin *et al.*, 1998 ▸; PERCAQ01, Barybin *et al.*, 2000 ▸), an 18-electron tantalum(I) cation in [Ta(CNX­yl)_7_]^+^[BF_4_]^−^ (WODNOS, Barybin *et al.*, 1999 ▸; WODNOS01, Barybin *et al.*, 2007 ▸), and a 15-electron vanadium(II) dication in [V(CN^t^Bu)_6_]^2+^[V(CO)_6_]^−^_2_ (ZEFXUD, Silverman *et al.*, 1981 ▸).

Uncharged halogen isocyanide mol­ecular complexes are the 14-electron V(CN^t^Bu)_3_Cl_3_ (CLBCNV, Silverman *et al.*, 1980 ▸; note that some carbon and hydrogen atoms are missing in the CIF taken from the CSD), three 15-electron complexes [V(CNX­yl)_4_I_2_](thf) (KAPKUH, Rehder *et al.*, 1999 ▸), [V(CN^t^Bu)_4_I_2_](thf)_2_ (ZASFOO, Böttcher *et al.*, 1995 ▸), [V(CN^t^Bu)_4_Br_2_](thf)_2_ (ZASFUU, Böttcher *et al.*, 1995 ▸), and 18-electron Ta(CNX­yl)_6_I (NEYVAP, Barybin *et al.*, 2007 ▸).

Two cationic halogen isocyanide complexes are known: a 15-electron vanadium(II) complex [V(CN^t^Bu)_5_I]^+^I^−^ (ZASFII, Böttcher *et al.*, 1995 ▸) and a 18-electron niobium(III) complex [Nb(CN^t^Bu)_6_I_2_]^+^I^−^(thf) (RARHOH, Collazo *et al.*, 1996 ▸).

## Synthesis and crystallization   

All synthetic manipulations were performed under vacuum or an atmosphere of purified argon, using Schlenk glassware, dry-box techniques and absolute solvents. [Et_4_N][V(CO)_6_] was recrystallized form a THF/Et_2_O mixture and dried under dynamic vacuum prior to use.

A solution of I_2_ (0.756 g, 2.98 mmol) in THF (45 ml) was dropwise added through a cannula to a cold (201 K) vigorously stirred solution of [Et_4_N][V(CO)_6_] (1.030 g, 2.95 mmol) in THF (65 ml), keeping the reaction mixture temperature below 203 K during addition. The resulting mixture was stirred for five minutes at 198 to 201 K. A solution of CNXyl (3.09 g, 23.6 mmol) in THF (50 ml) was added to the cold stirred reaction mixture, keeping its temperature below 208 K. The red reaction mixture was stirred overnight at 208 K. Then the mixture was allowed to warm up and filtered at room temperature. The white filter cake ([Et_4_N]I) was washed with THF (2 × 10 ml). All but *ca* 10 ml of THF was evaporated from the resulting solution under reduced pressure. Toluene (200 ml) was added to the residue, and the mixture was stirred at room temperature for several minutes. The dark-red precipitate was filtered off, washed with toluene (3 × 10 ml) and dried under dynamic vacuum. Red microcrystalline [V(CNX­yl)_7_]I was obtained in 54% yield (1.751 g, 1.60 mmol). IR (Nujol mull): ν_CN_ 2142 *w*, 2101 *m sh*, 2057 *m sh*, 2016 *vs br*, 1974 *s* cm^−1^.

Most toluene was evaporated from the remaining toluene solution to give previously studied green single crystals of [V(CNX­yl)_4_I_2_](thf). For its crystal and mol­ecular structure, see: Rehder *et al.* (1999 ▸).

A nearly saturated THF solution (at room temperature) of [V(CNX­yl)_7_]I (*ca* 20 ml) was placed into one ampoule of an H-shaped Schlenk vessel. Some reduced pressure was formed inside the vessel. Most solvent was slowly evaporated from the solution during eight hours into the second ampoule by cooling it with cold isopropyl alcohol (initial temperature was 223 K), producing several red single crystals of the title compound inside the first ampoule. The crystals were cut into smaller pieces prior to X-ray studies.

## Refinement details   

Crystal data, data collection and structure refinement details are summarized in Table 1 ▸. The hydrogen atoms were positioned geometrically (C—H distance = 0.950 Å for aromatic, 0.980 Å for methyl hydrogen atoms) and refined as riding atoms with *U*_iso_(H) = 1.2*U*_eq_(C) for aromatic and 1.5*U*_eq_(C) for methyl hydrogen atoms. A rotating group model was applied for all methyl groups.

## Supplementary Material

Crystal structure: contains datablock(s) I. DOI: 10.1107/S2056989015006015/wm5140sup1.cif

Structure factors: contains datablock(s) I. DOI: 10.1107/S2056989015006015/wm5140Isup2.hkl

Supporting information file. DOI: 10.1107/S2056989015006015/wm5140Isup3.cdx

CCDC reference: 1055983

Additional supporting information:  crystallographic information; 3D view; checkCIF report

## Figures and Tables

**Table 1:** Experimental details

Crystal data	
Chemical formula	[V(C_9_H_9_N)_7_]I
*M*_r_	1096.04
Crystal system, space group	Tetragonal, *P* 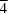 2_1_ *c*
Temperature (K)	173
*a*, *c* ()	22.765(2), 22.101(3)
*V* (^3^)	11454(2)
*Z*	8
Radiation type	Mo *K*
(mm^1^)	0.76
Crystal size (mm)	0.60 0.35 0.20

Data collection	
Diffractometer	Bruker SMART CCD area detector
Absorption correction	Multi-scan (*SADABS*; Bruker, 2003 ▸)
*T*_min_, *T*_max_	0.660, 0.863
No. of measured, independent and observed [*I* > 2(*I*)] reflections	81726, 10177, 8757
*R*_int_	0.047
(sin /)_max_ (^1^)	0.597

Refinement	
*R*[*F* ^2^ > 2(*F* ^2^)], *wR*(*F* ^2^), *S*	0.035, 0.079, 1.02
No. of reflections	10177
No. of parameters	664
H-atom treatment	H-atom parameters constrained
_max_, _min_ (e ^3^)	0.63, 0.55
Absolute structure	Flack (1983 ▸), 4655 Friedel pairs
Absolute structure parameter	0.002(14)

Computer programs: *SMART* and *SAINT* (Bruker, 2003 ▸), *SHELXS97*, *SHELXL97* and *SHELXTL* (Sheldrick, 2008 ▸) and *publCIF* (Westrip, 2010 ▸).

## supplementary crystallographic information

### Crystal data

**Table:**

[V(C_9_H_9_N)_7_]I	*D*_x_ = 1.271 Mg m^−^^3^
*M**_r_* = 1096.04	Mo *K*α radiation, λ = 0.71073 Å
Tetragonal, *P*42_1_*c*	Cell parameters from 7505 reflections
*a* = 22.765 (2) Å	θ = 2.6–25.0°
*c* = 22.101 (3) Å	µ = 0.76 mm^−^^1^
*V* = 11454 (2) Å^3^	*T* = 173 K
*Z* = 8	Block, red
*F*(000) = 4528	0.60 × 0.35 × 0.20 mm

### Data collection

**Table:**

Bruker SMART CCD area detector diffractometer	10177 independent reflections
Radiation source: fine-focus sealed tube	8757 reflections with *I* > 2σ(*I*)
Graphite monochromator	*R*_int_ = 0.047
phi and ω scans	θ_max_ = 25.1°, θ_min_ = 1.3°
Absorption correction: multi-scan (*SADABS*; Bruker, 2003)	*h* = −27→26
*T*_min_ = 0.660, *T*_max_ = 0.863	*k* = −27→27
81726 measured reflections	*l* = −25→26

### Refinement

**Table:**

Refinement on *F*^2^	Secondary atom site location: difference Fourier map
Least-squares matrix: full	Hydrogen site location: inferred from neighbouring sites
*R*[*F*^2^ > 2σ(*F*^2^)] = 0.035	H-atom parameters constrained
*wR*(*F*^2^) = 0.079	*w* = 1/[σ^2^(*F*_o_^2^) + (0.0345*P*)^2^ + 6.9436*P*] where *P* = (*F*_o_^2^ + 2*F*_c_^2^)/3
*S* = 1.02	(Δ/σ)_max_ = 0.001
10177 reflections	Δρ_max_ = 0.63 e Å^−^^3^
664 parameters	Δρ_min_ = −0.55 e Å^−^^3^
0 restraints	Absolute structure: Flack (1983), 4655 Friedel pairs
Primary atom site location: structure-invariant direct methods	Absolute structure parameter: −0.002 (14)

### Special details

**Table:**

Geometry. All e.s.d.'s (except the e.s.d. in the dihedral angle between two l.s. planes) are estimated using the full covariance matrix. The cell e.s.d.'s are taken into account individually in the estimation of e.s.d.'s in distances, angles and torsion angles; correlations between e.s.d.'s in cell parameters are only used when they are defined by crystal symmetry. An approximate (isotropic) treatment of cell e.s.d.'s is used for estimating e.s.d.'s involving l.s. planes.
Refinement. Refinement of *F*^2^ against ALL reflections. The weighted *R*-factor *wR* and goodness of fit *S* are based on *F*^2^, conventional *R*-factors *R* are based on *F*, with *F* set to zero for negative *F*^2^. The threshold expression of *F*^2^ > σ(*F*^2^) is used only for calculating *R*-factors(gt) *etc*. and is not relevant to the choice of reflections for refinement. *R*-factors based on *F*^2^ are statistically about twice as large as those based on *F*, and *R*- factors based on ALL data will be even larger.

### Fractional atomic coordinates and isotropic or equivalent isotropic displacement parameters (Å^2^)

**Table:**

	*x*	*y*	*z*	*U*_iso_*/*U*_eq_	
I1	0.0000	1.0000	0.268135 (13)	0.04485 (8)	
I2	0.0000	0.5000	0.312752 (14)	0.04651 (8)	
V1	0.24591 (2)	0.74322 (2)	0.55367 (2)	0.02709 (11)	
N1	0.36059 (12)	0.73626 (12)	0.63909 (12)	0.0345 (6)	
N2	0.16890 (13)	0.63043 (13)	0.52762 (12)	0.0359 (6)	
N3	0.33007 (13)	0.65308 (12)	0.48694 (12)	0.0379 (7)	
N4	0.30654 (12)	0.81424 (12)	0.44392 (14)	0.0383 (6)	
N5	0.20026 (13)	0.69516 (13)	0.68036 (13)	0.0395 (7)	
N6	0.13250 (14)	0.80208 (13)	0.49265 (15)	0.0470 (7)	
N7	0.23249 (12)	0.86458 (12)	0.63054 (13)	0.0360 (6)	
C1	0.32056 (14)	0.73978 (14)	0.60642 (15)	0.0337 (7)	
C2	0.40739 (14)	0.73143 (14)	0.68027 (14)	0.0335 (7)	
C3	0.43949 (16)	0.78138 (16)	0.69292 (17)	0.0470 (9)	
C4	0.48481 (19)	0.7760 (2)	0.73392 (19)	0.0610 (12)	
H4A	0.5076	0.8097	0.7437	0.073*	
C5	0.4977 (2)	0.7235 (2)	0.76078 (17)	0.0582 (10)	
H5A	0.5294	0.7210	0.7886	0.070*	
C6	0.46489 (18)	0.6741 (2)	0.74778 (15)	0.0486 (11)	
H6A	0.4740	0.6379	0.7669	0.058*	
C7	0.41828 (15)	0.67705 (15)	0.70664 (15)	0.0380 (8)	
C8	0.4240 (2)	0.83864 (19)	0.6635 (2)	0.0772 (15)	
H8A	0.4530	0.8685	0.6747	0.116*	
H8B	0.3849	0.8511	0.6770	0.116*	
H8C	0.4238	0.8337	0.6194	0.116*	
C9	0.38213 (19)	0.62423 (16)	0.6929 (2)	0.0556 (10)	
H9A	0.3853	0.6149	0.6497	0.083*	
H9B	0.3410	0.6322	0.7030	0.083*	
H9C	0.3963	0.5909	0.7168	0.083*	
C10	0.19707 (15)	0.67235 (16)	0.53512 (15)	0.0328 (8)	
C11	0.15327 (15)	0.57119 (15)	0.51994 (14)	0.0352 (8)	
C12	0.19218 (17)	0.52812 (17)	0.53980 (17)	0.0419 (9)	
C13	0.1782 (2)	0.47045 (18)	0.5278 (2)	0.0529 (11)	
H13A	0.2042	0.4403	0.5405	0.063*	
C14	0.12731 (19)	0.45542 (17)	0.4978 (2)	0.0570 (10)	
H14A	0.1188	0.4154	0.4895	0.068*	
C15	0.08923 (17)	0.4984 (2)	0.48008 (16)	0.0558 (9)	
H15A	0.0541	0.4877	0.4597	0.067*	
C16	0.10055 (15)	0.55774 (16)	0.49114 (18)	0.0429 (9)	
C17	0.24698 (18)	0.54464 (18)	0.5745 (2)	0.0623 (12)	
H17A	0.2659	0.5090	0.5899	0.093*	
H17B	0.2364	0.5702	0.6084	0.093*	
H17C	0.2742	0.5654	0.5476	0.093*	
C18	0.05718 (17)	0.60312 (18)	0.4730 (2)	0.0571 (11)	
H18A	0.0767	0.6414	0.4706	0.086*	
H18B	0.0256	0.6048	0.5031	0.086*	
H18C	0.0407	0.5930	0.4334	0.086*	
C19	0.29812 (14)	0.68541 (13)	0.51106 (15)	0.0310 (8)	
C20	0.37452 (15)	0.62325 (14)	0.45616 (14)	0.0331 (7)	
C21	0.36012 (16)	0.58963 (14)	0.40527 (15)	0.0386 (8)	
C22	0.40640 (18)	0.56365 (16)	0.37435 (17)	0.0478 (9)	
H22A	0.3984	0.5407	0.3394	0.057*	
C23	0.46327 (17)	0.57045 (16)	0.39317 (19)	0.0508 (10)	
H23A	0.4942	0.5529	0.3706	0.061*	
C24	0.47621 (16)	0.60236 (16)	0.44436 (18)	0.0484 (9)	
H24A	0.5159	0.6059	0.4572	0.058*	
C25	0.43235 (16)	0.62933 (16)	0.47723 (16)	0.0416 (8)	
C26	0.29740 (17)	0.58320 (19)	0.38533 (19)	0.0557 (10)	
H26A	0.2939	0.5494	0.3582	0.084*	
H26B	0.2850	0.6189	0.3640	0.084*	
H26C	0.2723	0.5772	0.4208	0.084*	
C27	0.44531 (18)	0.6660 (2)	0.53202 (19)	0.0595 (11)	
H27A	0.4878	0.6726	0.5350	0.089*	
H27B	0.4316	0.6455	0.5683	0.089*	
H27C	0.4251	0.7038	0.5285	0.089*	
C28	0.28413 (14)	0.78984 (14)	0.48403 (15)	0.0336 (7)	
C29	0.33785 (15)	0.84179 (15)	0.39740 (15)	0.0379 (8)	
C30	0.3369 (2)	0.90266 (17)	0.39382 (19)	0.0561 (11)	
C31	0.3725 (2)	0.9281 (2)	0.3494 (2)	0.0757 (15)	
H31A	0.3740	0.9696	0.3459	0.091*	
C32	0.4051 (2)	0.8944 (3)	0.3108 (3)	0.0823 (17)	
H32A	0.4288	0.9127	0.2810	0.099*	
C33	0.4039 (2)	0.8343 (2)	0.31477 (19)	0.0681 (13)	
H33A	0.4264	0.8115	0.2873	0.082*	
C34	0.37032 (16)	0.80637 (18)	0.35809 (16)	0.0480 (9)	
C35	0.2992 (2)	0.9377 (2)	0.4359 (3)	0.0824 (15)	
H35A	0.2579	0.9274	0.4296	0.124*	
H35B	0.3049	0.9797	0.4279	0.124*	
H35C	0.3103	0.9291	0.4778	0.124*	
C36	0.37023 (19)	0.74102 (18)	0.36375 (18)	0.0538 (10)	
H36A	0.3912	0.7238	0.3293	0.081*	
H36B	0.3296	0.7267	0.3642	0.081*	
H36C	0.3899	0.7297	0.4014	0.081*	
C37	0.21572 (14)	0.71260 (14)	0.63360 (15)	0.0334 (7)	
C38	0.18414 (16)	0.68108 (16)	0.73982 (15)	0.0378 (8)	
C39	0.13717 (17)	0.64340 (18)	0.74923 (16)	0.0441 (9)	
C40	0.12119 (18)	0.63289 (17)	0.8089 (2)	0.0526 (10)	
H40A	0.0891	0.6076	0.8175	0.063*	
C41	0.1512 (2)	0.65865 (19)	0.8557 (2)	0.0599 (12)	
H41A	0.1391	0.6515	0.8962	0.072*	
C42	0.19816 (19)	0.69438 (18)	0.84504 (17)	0.0515 (10)	
H42A	0.2186	0.7113	0.8782	0.062*	
C43	0.21659 (16)	0.70640 (16)	0.78663 (15)	0.0419 (8)	
C44	0.10581 (18)	0.6151 (2)	0.6972 (2)	0.0645 (12)	
H44A	0.0889	0.6455	0.6711	0.097*	
H44B	0.0744	0.5898	0.7126	0.097*	
H44C	0.1337	0.5914	0.6737	0.097*	
C45	0.26886 (19)	0.7437 (2)	0.77305 (19)	0.0587 (10)	
H45A	0.2864	0.7573	0.8110	0.088*	
H45B	0.2567	0.7777	0.7489	0.088*	
H45C	0.2978	0.7206	0.7504	0.088*	
C46	0.17313 (16)	0.77903 (14)	0.51357 (14)	0.0363 (8)	
C47	0.08296 (16)	0.83284 (17)	0.47315 (17)	0.0432 (9)	
C48	0.0383 (2)	0.8439 (2)	0.5139 (2)	0.0655 (13)	
C49	−0.0087 (2)	0.8771 (3)	0.4928 (3)	0.105 (2)	
H49A	−0.0402	0.8863	0.5193	0.126*	
C50	−0.0101 (3)	0.8965 (3)	0.4348 (3)	0.114 (2)	
H50A	−0.0428	0.9189	0.4214	0.137*	
C51	0.0338 (3)	0.8848 (2)	0.3958 (2)	0.0849 (17)	
H51A	0.0312	0.8985	0.3553	0.102*	
C52	0.0821 (2)	0.85342 (17)	0.41383 (17)	0.0533 (11)	
C53	0.0428 (3)	0.8235 (3)	0.5781 (2)	0.103 (2)	
H53A	0.0498	0.7811	0.5789	0.154*	
H53B	0.0062	0.8325	0.5995	0.154*	
H53C	0.0756	0.8438	0.5980	0.154*	
C54	0.1317 (2)	0.8423 (2)	0.3714 (2)	0.0801 (15)	
H54A	0.1270	0.8670	0.3353	0.120*	
H54B	0.1318	0.8009	0.3595	0.120*	
H54C	0.1689	0.8519	0.3914	0.120*	
C55	0.23719 (14)	0.82285 (14)	0.60144 (14)	0.0345 (7)	
C56	0.22834 (15)	0.91500 (14)	0.66724 (14)	0.0347 (8)	
C57	0.18251 (16)	0.91861 (16)	0.70904 (15)	0.0400 (8)	
C58	0.1798 (2)	0.9686 (2)	0.74417 (18)	0.0558 (12)	
H58A	0.1490	0.9732	0.7728	0.067*	
C59	0.2208 (2)	1.0113 (2)	0.7381 (2)	0.0666 (14)	
H59A	0.2184	1.0451	0.7632	0.080*	
C60	0.2660 (2)	1.00723 (19)	0.69679 (19)	0.0635 (11)	
H60A	0.2940	1.0380	0.6937	0.076*	
C61	0.27067 (18)	0.95816 (17)	0.65963 (17)	0.0493 (10)	
C62	0.13768 (18)	0.8711 (2)	0.7169 (2)	0.0597 (11)	
H62A	0.1354	0.8600	0.7596	0.090*	
H62B	0.1490	0.8368	0.6927	0.090*	
H62C	0.0993	0.8854	0.7033	0.090*	
C63	0.3188 (2)	0.9542 (2)	0.6138 (2)	0.0766 (14)	
H63A	0.3280	0.9128	0.6061	0.115*	
H63B	0.3539	0.9742	0.6292	0.115*	
H63C	0.3061	0.9729	0.5761	0.115*	

### Atomic displacement parameters (Å^2^)

**Table:**

	*U*^11^	*U*^22^	*U*^33^	*U*^12^	*U*^13^	*U*^23^
I1	0.05314 (19)	0.04626 (18)	0.03514 (15)	0.0039 (2)	0.000	0.000
I2	0.03786 (16)	0.05501 (18)	0.04666 (17)	0.00683 (17)	0.000	0.000
V1	0.0307 (3)	0.0269 (3)	0.0236 (2)	−0.0003 (2)	−0.0009 (2)	−0.0013 (2)
N1	0.0376 (16)	0.0347 (15)	0.0313 (15)	0.0023 (13)	−0.0026 (13)	−0.0044 (12)
N2	0.0390 (16)	0.0391 (17)	0.0296 (15)	−0.0026 (14)	−0.0014 (12)	0.0029 (12)
N3	0.0428 (17)	0.0365 (16)	0.034 (2)	0.0044 (14)	0.0025 (12)	−0.0009 (12)
N4	0.0433 (16)	0.0432 (17)	0.0282 (15)	−0.0006 (13)	0.0017 (14)	−0.0002 (14)
N5	0.0429 (16)	0.0430 (16)	0.0327 (17)	−0.0026 (13)	0.0016 (14)	0.0005 (14)
N6	0.0494 (18)	0.0392 (16)	0.052 (2)	0.0027 (14)	−0.0146 (16)	0.0060 (15)
N7	0.0428 (16)	0.0335 (15)	0.0316 (15)	0.0014 (13)	0.0034 (12)	−0.0007 (13)
C1	0.0408 (19)	0.0314 (17)	0.0289 (17)	−0.0003 (15)	0.0042 (15)	−0.0033 (14)
C2	0.0327 (17)	0.0440 (19)	0.0239 (17)	0.0018 (15)	0.0010 (13)	−0.0037 (14)
C3	0.052 (2)	0.052 (2)	0.036 (2)	−0.0121 (18)	0.0012 (18)	−0.0058 (17)
C4	0.062 (3)	0.078 (3)	0.042 (2)	−0.017 (2)	−0.005 (2)	−0.020 (2)
C5	0.045 (2)	0.089 (3)	0.0414 (19)	0.011 (3)	−0.008 (3)	−0.012 (2)
C6	0.048 (2)	0.066 (3)	0.031 (2)	0.020 (2)	−0.0001 (15)	0.0045 (17)
C7	0.0408 (19)	0.044 (2)	0.0293 (18)	0.0070 (16)	0.0057 (14)	−0.0009 (15)
C8	0.111 (4)	0.047 (3)	0.074 (3)	−0.022 (3)	−0.013 (3)	0.001 (2)
C9	0.069 (3)	0.039 (2)	0.059 (2)	−0.0055 (19)	0.005 (2)	0.0039 (19)
C10	0.0349 (18)	0.038 (2)	0.0255 (18)	−0.0025 (17)	−0.0010 (14)	0.0041 (14)
C11	0.0385 (19)	0.0396 (19)	0.0275 (18)	−0.0096 (16)	0.0007 (14)	0.0024 (14)
C12	0.045 (2)	0.045 (2)	0.036 (2)	−0.0044 (17)	−0.0067 (16)	0.0104 (16)
C13	0.064 (3)	0.038 (2)	0.057 (3)	−0.005 (2)	−0.005 (2)	0.0139 (19)
C14	0.077 (3)	0.039 (2)	0.055 (2)	−0.015 (2)	−0.003 (3)	0.004 (2)
C15	0.057 (2)	0.056 (2)	0.054 (2)	−0.022 (3)	−0.0118 (16)	0.007 (3)
C16	0.041 (2)	0.045 (2)	0.043 (2)	−0.0074 (16)	0.0003 (18)	0.0064 (18)
C17	0.058 (3)	0.053 (2)	0.076 (3)	−0.003 (2)	−0.026 (2)	0.017 (2)
C18	0.039 (2)	0.059 (3)	0.074 (3)	−0.002 (2)	−0.0104 (19)	0.014 (2)
C19	0.0340 (17)	0.0283 (17)	0.031 (2)	−0.0014 (15)	−0.0039 (14)	0.0005 (13)
C20	0.0376 (18)	0.0294 (17)	0.0323 (19)	0.0053 (14)	0.0048 (14)	0.0028 (13)
C21	0.047 (2)	0.0309 (18)	0.038 (2)	0.0073 (16)	−0.0025 (16)	−0.0020 (15)
C22	0.065 (3)	0.040 (2)	0.039 (2)	0.0068 (18)	0.0034 (18)	−0.0064 (16)
C23	0.053 (2)	0.042 (2)	0.057 (3)	0.0152 (18)	0.012 (2)	−0.0009 (19)
C24	0.0407 (19)	0.049 (2)	0.055 (2)	0.0071 (16)	0.0029 (18)	−0.0055 (19)
C25	0.046 (2)	0.043 (2)	0.0351 (19)	0.0054 (18)	−0.0010 (15)	0.0003 (15)
C26	0.057 (3)	0.062 (3)	0.048 (2)	0.005 (2)	−0.014 (2)	−0.009 (2)
C27	0.051 (2)	0.080 (3)	0.047 (2)	0.000 (2)	−0.0006 (19)	−0.016 (2)
C28	0.0370 (19)	0.0343 (18)	0.0293 (18)	0.0048 (15)	−0.0025 (14)	−0.0034 (14)
C29	0.0404 (19)	0.046 (2)	0.0273 (17)	−0.0083 (15)	−0.0071 (15)	0.0086 (15)
C30	0.071 (3)	0.047 (2)	0.050 (2)	−0.007 (2)	−0.018 (2)	0.0131 (19)
C31	0.101 (4)	0.059 (3)	0.067 (3)	−0.027 (3)	−0.024 (3)	0.031 (3)
C32	0.086 (4)	0.110 (5)	0.050 (3)	−0.037 (3)	0.001 (3)	0.028 (3)
C33	0.068 (3)	0.100 (4)	0.036 (2)	−0.014 (3)	0.009 (2)	0.013 (2)
C34	0.045 (2)	0.071 (3)	0.0272 (19)	−0.0097 (19)	−0.0018 (16)	0.0037 (18)
C35	0.108 (4)	0.051 (3)	0.087 (4)	0.020 (3)	−0.016 (3)	0.001 (3)
C36	0.062 (2)	0.058 (3)	0.041 (2)	0.006 (2)	0.0020 (19)	−0.0055 (19)
C37	0.0378 (19)	0.0358 (18)	0.0267 (18)	−0.0015 (14)	−0.0013 (14)	−0.0004 (14)
C38	0.043 (2)	0.040 (2)	0.0299 (17)	0.0090 (15)	0.0067 (16)	0.0048 (16)
C39	0.040 (2)	0.052 (3)	0.041 (2)	0.0081 (17)	0.0056 (15)	0.0107 (16)
C40	0.053 (2)	0.051 (2)	0.054 (2)	0.0049 (19)	0.015 (2)	0.013 (2)
C41	0.079 (3)	0.061 (3)	0.040 (2)	0.015 (2)	0.017 (2)	0.010 (2)
C42	0.065 (3)	0.055 (2)	0.034 (2)	0.014 (2)	0.0021 (19)	−0.0038 (18)
C43	0.048 (2)	0.047 (2)	0.0310 (19)	0.0097 (17)	0.0032 (15)	−0.0021 (15)
C44	0.048 (2)	0.076 (3)	0.069 (3)	−0.021 (2)	0.003 (2)	0.004 (3)
C45	0.065 (3)	0.066 (3)	0.046 (2)	−0.004 (2)	−0.0022 (19)	−0.012 (2)
C46	0.044 (2)	0.0310 (17)	0.034 (2)	−0.0023 (16)	−0.0044 (15)	0.0044 (14)
C47	0.042 (2)	0.048 (2)	0.040 (2)	0.0003 (18)	−0.0146 (16)	0.0049 (16)
C48	0.055 (3)	0.094 (3)	0.048 (3)	−0.007 (2)	−0.008 (2)	−0.003 (2)
C49	0.056 (3)	0.168 (6)	0.091 (4)	0.034 (4)	−0.011 (4)	−0.039 (4)
C50	0.094 (5)	0.146 (6)	0.102 (5)	0.070 (4)	−0.055 (4)	−0.024 (4)
C51	0.118 (4)	0.083 (4)	0.054 (3)	0.036 (3)	−0.044 (3)	−0.001 (3)
C52	0.076 (3)	0.045 (2)	0.038 (2)	0.009 (2)	−0.015 (2)	−0.0031 (17)
C53	0.100 (4)	0.155 (6)	0.053 (3)	−0.049 (4)	0.010 (3)	0.014 (3)
C54	0.111 (4)	0.087 (4)	0.043 (3)	0.005 (3)	0.011 (3)	−0.004 (2)
C55	0.0384 (19)	0.0357 (18)	0.0295 (18)	0.0005 (15)	0.0042 (14)	0.0033 (15)
C56	0.050 (2)	0.0285 (17)	0.0251 (17)	0.0064 (15)	−0.0068 (15)	−0.0016 (13)
C57	0.042 (2)	0.045 (2)	0.0323 (19)	0.0097 (16)	−0.0055 (15)	−0.0071 (15)
C58	0.062 (3)	0.058 (3)	0.047 (3)	0.028 (3)	−0.0029 (19)	−0.0160 (19)
C59	0.091 (4)	0.049 (3)	0.060 (3)	0.019 (3)	−0.015 (2)	−0.023 (2)
C60	0.091 (3)	0.041 (2)	0.059 (2)	−0.013 (2)	−0.014 (2)	0.001 (2)
C61	0.068 (3)	0.045 (2)	0.035 (2)	−0.0026 (19)	−0.0012 (18)	0.0061 (16)
C62	0.047 (2)	0.079 (3)	0.053 (3)	−0.009 (2)	0.0072 (19)	−0.012 (2)
C63	0.095 (4)	0.073 (3)	0.062 (3)	−0.024 (3)	0.025 (3)	0.006 (2)

### Geometric parameters (Å, º)

**Table:**

V1—C10	2.002 (4)	C27—H27B	0.9800
V1—C19	2.008 (3)	C27—H27C	0.9800
V1—C37	2.020 (3)	C29—C30	1.388 (5)
V1—C46	2.048 (3)	C29—C34	1.397 (5)
V1—C28	2.062 (4)	C30—C31	1.398 (6)
V1—C1	2.062 (3)	C30—C35	1.497 (7)
V1—C55	2.107 (3)	C31—C32	1.366 (8)
N1—C1	1.165 (4)	C31—H31A	0.9500
N1—C2	1.406 (4)	C32—C33	1.371 (7)
N2—C10	1.162 (4)	C32—H32A	0.9500
N2—C11	1.405 (4)	C33—C34	1.379 (5)
N3—C19	1.164 (4)	C33—H33A	0.9500
N3—C20	1.396 (4)	C34—C36	1.493 (6)
N4—C28	1.164 (4)	C35—H35A	0.9800
N4—C29	1.399 (4)	C35—H35B	0.9800
N5—C37	1.162 (4)	C35—H35C	0.9800
N5—C38	1.401 (4)	C36—H36A	0.9800
N6—C46	1.160 (4)	C36—H36B	0.9800
N6—C47	1.396 (4)	C36—H36C	0.9800
N7—C55	1.152 (4)	C38—C39	1.387 (5)
N7—C56	1.409 (4)	C38—C43	1.396 (5)
C2—C3	1.380 (5)	C39—C40	1.390 (6)
C2—C7	1.391 (5)	C39—C44	1.500 (6)
C3—C4	1.378 (5)	C40—C41	1.371 (6)
C3—C8	1.499 (6)	C40—H40A	0.9500
C4—C5	1.367 (6)	C41—C42	1.363 (6)
C4—H4A	0.9500	C41—H41A	0.9500
C5—C6	1.380 (6)	C42—C43	1.385 (5)
C5—H5A	0.9500	C42—H42A	0.9500
C6—C7	1.399 (5)	C43—C45	1.492 (6)
C6—H6A	0.9500	C44—H44A	0.9800
C7—C9	1.488 (5)	C44—H44B	0.9800
C8—H8A	0.9800	C44—H44C	0.9800
C8—H8B	0.9800	C45—H45A	0.9800
C8—H8C	0.9800	C45—H45B	0.9800
C9—H9A	0.9800	C45—H45C	0.9800
C9—H9B	0.9800	C47—C48	1.382 (6)
C9—H9C	0.9800	C47—C52	1.392 (5)
C11—C12	1.392 (5)	C48—C49	1.390 (7)
C11—C16	1.393 (5)	C48—C53	1.497 (7)
C12—C13	1.377 (5)	C49—C50	1.355 (8)
C12—C17	1.512 (5)	C49—H49A	0.9500
C13—C14	1.377 (6)	C50—C51	1.345 (8)
C13—H13A	0.9500	C50—H50A	0.9500
C14—C15	1.366 (6)	C51—C52	1.371 (6)
C14—H14A	0.9500	C51—H51A	0.9500
C15—C16	1.396 (6)	C52—C54	1.489 (6)
C15—H15A	0.9500	C53—H53A	0.9800
C16—C18	1.484 (5)	C53—H53B	0.9800
C17—H17A	0.9800	C53—H53C	0.9800
C17—H17B	0.9800	C54—H54A	0.9800
C17—H17C	0.9800	C54—H54B	0.9800
C18—H18A	0.9800	C54—H54C	0.9800
C18—H18B	0.9800	C56—C61	1.386 (5)
C18—H18C	0.9800	C56—C57	1.396 (5)
C20—C21	1.399 (5)	C57—C58	1.380 (5)
C20—C25	1.403 (5)	C57—C62	1.497 (5)
C21—C22	1.388 (5)	C58—C59	1.354 (6)
C21—C26	1.501 (5)	C58—H58A	0.9500
C22—C23	1.368 (5)	C59—C60	1.379 (7)
C22—H22A	0.9500	C59—H59A	0.9500
C23—C24	1.376 (6)	C60—C61	1.391 (6)
C23—H23A	0.9500	C60—H60A	0.9500
C24—C25	1.379 (5)	C61—C63	1.495 (6)
C24—H24A	0.9500	C62—H62A	0.9800
C25—C27	1.500 (5)	C62—H62B	0.9800
C26—H26A	0.9800	C62—H62C	0.9800
C26—H26B	0.9800	C63—H63A	0.9800
C26—H26C	0.9800	C63—H63B	0.9800
C27—H27A	0.9800	C63—H63C	0.9800
			
C10—V1—C19	72.81 (13)	C30—C29—N4	118.8 (3)
C10—V1—C37	73.26 (13)	C34—C29—N4	117.9 (3)
C19—V1—C37	112.67 (13)	C29—C30—C31	116.4 (4)
C10—V1—C46	77.46 (14)	C29—C30—C35	120.4 (4)
C19—V1—C46	122.46 (14)	C31—C30—C35	123.2 (4)
C37—V1—C46	103.92 (13)	C32—C31—C30	121.4 (4)
C10—V1—C28	119.75 (13)	C32—C31—H31A	119.3
C19—V1—C28	74.78 (12)	C30—C31—H31A	119.3
C37—V1—C28	166.93 (13)	C31—C32—C33	120.6 (5)
C46—V1—C28	79.25 (13)	C31—C32—H32A	119.7
C10—V1—C1	122.88 (13)	C33—C32—H32A	119.7
C19—V1—C1	75.66 (13)	C32—C33—C34	121.0 (5)
C37—V1—C1	76.87 (13)	C32—C33—H33A	119.5
C46—V1—C1	157.87 (13)	C34—C33—H33A	119.5
C28—V1—C1	95.38 (13)	C33—C34—C29	117.3 (4)
C10—V1—C55	138.05 (14)	C33—C34—C36	121.2 (4)
C19—V1—C55	148.71 (13)	C29—C34—C36	121.5 (3)
C37—V1—C55	80.02 (12)	C30—C35—H35A	109.5
C46—V1—C55	78.36 (13)	C30—C35—H35B	109.5
C28—V1—C55	88.33 (12)	H35A—C35—H35B	109.5
C1—V1—C55	80.05 (12)	C30—C35—H35C	109.5
C1—N1—C2	177.8 (3)	H35A—C35—H35C	109.5
C10—N2—C11	161.0 (3)	H35B—C35—H35C	109.5
C19—N3—C20	169.7 (3)	C34—C36—H36A	109.5
C28—N4—C29	175.3 (3)	C34—C36—H36B	109.5
C37—N5—C38	172.6 (3)	H36A—C36—H36B	109.5
C46—N6—C47	174.1 (4)	C34—C36—H36C	109.5
C55—N7—C56	178.1 (4)	H36A—C36—H36C	109.5
N1—C1—V1	175.7 (3)	H36B—C36—H36C	109.5
C3—C2—C7	123.7 (3)	N5—C37—V1	177.7 (3)
C3—C2—N1	117.9 (3)	C39—C38—C43	123.5 (3)
C7—C2—N1	118.4 (3)	C39—C38—N5	119.0 (3)
C4—C3—C2	117.2 (4)	C43—C38—N5	117.5 (3)
C4—C3—C8	122.6 (4)	C38—C39—C40	116.8 (4)
C2—C3—C8	120.2 (4)	C38—C39—C44	121.2 (3)
C5—C4—C3	121.5 (4)	C40—C39—C44	122.0 (4)
C5—C4—H4A	119.2	C41—C40—C39	120.8 (4)
C3—C4—H4A	119.2	C41—C40—H40A	119.6
C4—C5—C6	120.5 (4)	C39—C40—H40A	119.6
C4—C5—H5A	119.8	C42—C41—C40	121.0 (4)
C6—C5—H5A	119.8	C42—C41—H41A	119.5
C5—C6—C7	120.4 (4)	C40—C41—H41A	119.5
C5—C6—H6A	119.8	C41—C42—C43	121.1 (4)
C7—C6—H6A	119.8	C41—C42—H42A	119.4
C2—C7—C6	116.8 (3)	C43—C42—H42A	119.4
C2—C7—C9	122.3 (3)	C42—C43—C38	116.7 (4)
C6—C7—C9	120.9 (4)	C42—C43—C45	122.8 (4)
C3—C8—H8A	109.5	C38—C43—C45	120.5 (3)
C3—C8—H8B	109.5	C39—C44—H44A	109.5
H8A—C8—H8B	109.5	C39—C44—H44B	109.5
C3—C8—H8C	109.5	H44A—C44—H44B	109.5
H8A—C8—H8C	109.5	C39—C44—H44C	109.5
H8B—C8—H8C	109.5	H44A—C44—H44C	109.5
C7—C9—H9A	109.5	H44B—C44—H44C	109.5
C7—C9—H9B	109.5	C43—C45—H45A	109.5
H9A—C9—H9B	109.5	C43—C45—H45B	109.5
C7—C9—H9C	109.5	H45A—C45—H45B	109.5
H9A—C9—H9C	109.5	C43—C45—H45C	109.5
H9B—C9—H9C	109.5	H45A—C45—H45C	109.5
N2—C10—V1	176.3 (3)	H45B—C45—H45C	109.5
C12—C11—C16	122.5 (3)	N6—C46—V1	176.3 (3)
C12—C11—N2	118.5 (3)	C48—C47—C52	122.8 (4)
C16—C11—N2	119.0 (3)	C48—C47—N6	119.0 (3)
C13—C12—C11	117.6 (4)	C52—C47—N6	118.1 (4)
C13—C12—C17	121.7 (3)	C47—C48—C49	116.5 (4)
C11—C12—C17	120.6 (3)	C47—C48—C53	120.7 (4)
C12—C13—C14	121.6 (4)	C49—C48—C53	122.7 (5)
C12—C13—H13A	119.2	C50—C49—C48	120.8 (5)
C14—C13—H13A	119.2	C50—C49—H49A	119.6
C15—C14—C13	119.6 (4)	C48—C49—H49A	119.6
C15—C14—H14A	120.2	C51—C50—C49	121.6 (5)
C13—C14—H14A	120.2	C51—C50—H50A	119.2
C14—C15—C16	121.7 (4)	C49—C50—H50A	119.2
C14—C15—H15A	119.1	C50—C51—C52	120.9 (5)
C16—C15—H15A	119.1	C50—C51—H51A	119.6
C11—C16—C15	116.9 (3)	C52—C51—H51A	119.6
C11—C16—C18	122.9 (3)	C51—C52—C47	117.3 (4)
C15—C16—C18	120.2 (3)	C51—C52—C54	120.9 (4)
C12—C17—H17A	109.5	C47—C52—C54	121.7 (4)
C12—C17—H17B	109.5	C48—C53—H53A	109.5
H17A—C17—H17B	109.5	C48—C53—H53B	109.5
C12—C17—H17C	109.5	H53A—C53—H53B	109.5
H17A—C17—H17C	109.5	C48—C53—H53C	109.5
H17B—C17—H17C	109.5	H53A—C53—H53C	109.5
C16—C18—H18A	109.5	H53B—C53—H53C	109.5
C16—C18—H18B	109.5	C52—C54—H54A	109.5
H18A—C18—H18B	109.5	C52—C54—H54B	109.5
C16—C18—H18C	109.5	H54A—C54—H54B	109.5
H18A—C18—H18C	109.5	C52—C54—H54C	109.5
H18B—C18—H18C	109.5	H54A—C54—H54C	109.5
N3—C19—V1	177.6 (3)	H54B—C54—H54C	109.5
N3—C20—C21	119.2 (3)	N7—C55—V1	176.1 (3)
N3—C20—C25	118.1 (3)	C61—C56—C57	123.9 (3)
C21—C20—C25	122.7 (3)	C61—C56—N7	117.4 (3)
C22—C21—C20	116.8 (3)	C57—C56—N7	118.6 (3)
C22—C21—C26	122.4 (3)	C58—C57—C56	117.0 (4)
C20—C21—C26	120.8 (3)	C58—C57—C62	120.1 (4)
C23—C22—C21	121.3 (4)	C56—C57—C62	122.9 (3)
C23—C22—H22A	119.3	C59—C58—C57	120.4 (4)
C21—C22—H22A	119.3	C59—C58—H58A	119.8
C22—C23—C24	120.8 (4)	C57—C58—H58A	119.8
C22—C23—H23A	119.6	C58—C59—C60	122.2 (4)
C24—C23—H23A	119.6	C58—C59—H59A	118.9
C23—C24—C25	120.9 (4)	C60—C59—H59A	118.9
C23—C24—H24A	119.6	C59—C60—C61	120.1 (4)
C25—C24—H24A	119.6	C59—C60—H60A	119.9
C24—C25—C20	117.4 (3)	C61—C60—H60A	119.9
C24—C25—C27	122.0 (3)	C56—C61—C60	116.4 (4)
C20—C25—C27	120.5 (3)	C56—C61—C63	123.3 (4)
C21—C26—H26A	109.5	C60—C61—C63	120.3 (4)
C21—C26—H26B	109.5	C57—C62—H62A	109.5
H26A—C26—H26B	109.5	C57—C62—H62B	109.5
C21—C26—H26C	109.5	H62A—C62—H62B	109.5
H26A—C26—H26C	109.5	C57—C62—H62C	109.5
H26B—C26—H26C	109.5	H62A—C62—H62C	109.5
C25—C27—H27A	109.5	H62B—C62—H62C	109.5
C25—C27—H27B	109.5	C61—C63—H63A	109.5
H27A—C27—H27B	109.5	C61—C63—H63B	109.5
C25—C27—H27C	109.5	H63A—C63—H63B	109.5
H27A—C27—H27C	109.5	C61—C63—H63C	109.5
H27B—C27—H27C	109.5	H63A—C63—H63C	109.5
N4—C28—V1	177.5 (3)	H63B—C63—H63C	109.5
C30—C29—C34	123.3 (4)		
			
C7—C2—C3—C4	0.3 (5)	C35—C30—C31—C32	−178.3 (5)
N1—C2—C3—C4	179.3 (3)	C30—C31—C32—C33	−0.1 (8)
C7—C2—C3—C8	−178.6 (4)	C31—C32—C33—C34	−0.8 (8)
N1—C2—C3—C8	0.4 (5)	C32—C33—C34—C29	0.1 (6)
C2—C3—C4—C5	0.3 (6)	C32—C33—C34—C36	−178.2 (4)
C8—C3—C4—C5	179.1 (4)	C30—C29—C34—C33	1.5 (5)
C3—C4—C5—C6	−0.6 (6)	N4—C29—C34—C33	−176.1 (3)
C4—C5—C6—C7	0.4 (6)	C30—C29—C34—C36	179.7 (4)
C3—C2—C7—C6	−0.4 (5)	N4—C29—C34—C36	2.1 (5)
N1—C2—C7—C6	−179.4 (3)	C43—C38—C39—C40	2.5 (6)
C3—C2—C7—C9	178.8 (3)	N5—C38—C39—C40	−177.1 (3)
N1—C2—C7—C9	−0.2 (5)	C43—C38—C39—C44	−176.9 (4)
C5—C6—C7—C2	0.1 (5)	N5—C38—C39—C44	3.5 (6)
C5—C6—C7—C9	−179.2 (4)	C38—C39—C40—C41	−0.4 (6)
C10—N2—C11—C12	20.2 (11)	C44—C39—C40—C41	179.0 (4)
C10—N2—C11—C16	−157.7 (9)	C39—C40—C41—C42	−1.2 (6)
C16—C11—C12—C13	2.7 (6)	C40—C41—C42—C43	0.8 (6)
N2—C11—C12—C13	−175.2 (4)	C41—C42—C43—C38	1.1 (6)
C16—C11—C12—C17	−176.0 (4)	C41—C42—C43—C45	−178.0 (4)
N2—C11—C12—C17	6.1 (5)	C39—C38—C43—C42	−2.9 (6)
C11—C12—C13—C14	−0.7 (7)	N5—C38—C43—C42	176.8 (3)
C17—C12—C13—C14	178.0 (4)	C39—C38—C43—C45	176.3 (4)
C12—C13—C14—C15	−0.9 (7)	N5—C38—C43—C45	−4.0 (5)
C13—C14—C15—C16	0.4 (6)	C52—C47—C48—C49	0.1 (7)
C12—C11—C16—C15	−3.0 (5)	N6—C47—C48—C49	177.0 (4)
N2—C11—C16—C15	174.8 (3)	C52—C47—C48—C53	−176.6 (5)
C12—C11—C16—C18	176.3 (4)	N6—C47—C48—C53	0.3 (7)
N2—C11—C16—C18	−5.8 (5)	C47—C48—C49—C50	0.9 (9)
C14—C15—C16—C11	1.4 (6)	C53—C48—C49—C50	177.6 (6)
C14—C15—C16—C18	−177.9 (4)	C48—C49—C50—C51	−0.4 (11)
C19—N3—C20—C21	−124.1 (17)	C49—C50—C51—C52	−1.0 (10)
C19—N3—C20—C25	54.8 (19)	C50—C51—C52—C47	1.9 (8)
N3—C20—C21—C22	176.4 (3)	C50—C51—C52—C54	−177.7 (5)
C25—C20—C21—C22	−2.4 (5)	C48—C47—C52—C51	−1.5 (6)
N3—C20—C21—C26	−2.8 (5)	N6—C47—C52—C51	−178.4 (4)
C25—C20—C21—C26	178.5 (3)	C48—C47—C52—C54	178.1 (4)
C20—C21—C22—C23	0.4 (5)	N6—C47—C52—C54	1.2 (6)
C26—C21—C22—C23	179.6 (4)	C61—C56—C57—C58	0.4 (5)
C21—C22—C23—C24	1.4 (6)	N7—C56—C57—C58	−180.0 (3)
C22—C23—C24—C25	−1.4 (6)	C61—C56—C57—C62	−179.3 (4)
C23—C24—C25—C20	−0.5 (5)	N7—C56—C57—C62	0.3 (5)
C23—C24—C25—C27	−178.0 (4)	C56—C57—C58—C59	−0.9 (6)
N3—C20—C25—C24	−176.4 (3)	C62—C57—C58—C59	178.8 (4)
C21—C20—C25—C24	2.4 (5)	C57—C58—C59—C60	0.8 (7)
N3—C20—C25—C27	1.1 (5)	C58—C59—C60—C61	−0.1 (7)
C21—C20—C25—C27	179.9 (3)	C57—C56—C61—C60	0.3 (5)
C34—C29—C30—C31	−2.3 (6)	N7—C56—C61—C60	−179.3 (3)
N4—C29—C30—C31	175.3 (3)	C57—C56—C61—C63	−178.8 (4)
C34—C29—C30—C35	177.5 (4)	N7—C56—C61—C63	1.6 (5)
N4—C29—C30—C35	−4.9 (6)	C59—C60—C61—C56	−0.5 (6)
C29—C30—C31—C32	1.5 (7)	C59—C60—C61—C63	178.6 (4)
